# Founders’ flow: A qualitative study on the role of flow experience in early start-up stages

**DOI:** 10.1371/journal.pone.0292580

**Published:** 2023-10-05

**Authors:** Leonie Kloep, Katharina Roese, Corinna Peifer

**Affiliations:** 1 Department of Psychology, Research Group Work and Health, University of Lübeck, Lübeck, Germany; 2 Department of Occupational Therapy, Institute of Health Sciences, University of Lübeck, Lübeck, Germany; National University of Modern Languages, PAKISTAN

## Abstract

Flow experience is a state of complete absorption while performing an optimally challenging and enjoyable task. It is often experienced at work—both in the form of individual and team flow—and can have a positive effect on performance and well-being. However, start-up founders’ work situation differs from that in established companies, facing not only great autonomy but also new challenges, uncertainty, and risks. It can be assumed that flow also provides benefits in start-ups, however, this has not yet been examined in depth and the factors that may operate differently in start-ups in comparison to other work contexts have not yet been explored in detail. Using a qualitative research approach, 21 founders from different industries were interviewed. Enhancing and inhibiting factors of flow and team flow as well as consequences for the founders and the start-up in general were examined and structured with the help of a qualitative content analysis. A variety of contexts was identified in which founders experienced flow and team flow. Various factors on the individual, task-related and organizational sphere were found to be perceived as promoting or hindering flow and team flow, e.g. well-being, autonomy or the environment. The findings regarding the consequences of flow and team flow show that these mainly are very desirable states for founders, e.g. leading to better results, progress or team processes. Only few negative consequences were identified, e.g. perfectionism. Thus, it is helpful to foster flow in the start-up context. Possible approaches derived from the participants’ statements to this could be, for example, to design flow-promoting environments or participation in specific workshops.

## Introduction

A team of young students decides to found their own start-up after graduation. They invest a lot of time in their idea and team members work passionately for the common goal. While people around them mainly perceive the uncertainties in starting a business and wonder if it is worth it, the team members do not lose their motivation. They seem to be absorbed by their work; nothing can stop them when they are working on their idea.

Starting a business is a process fraught with risks, difficult decisions, and uncertainty [1, 2]. Many factors are unpredictable and the path to a functioning start-up is a series of challenging ups and downs [1]. Often start-ups have not yet developed sufficient resilience in the team to face challenges with confidence [3]. Nevertheless, many founders seem to be absorbed in their task, do not lose their vision, and motivate themselves every day anew, although the work in many cases does not yet bring them any financial or other rewards.

One possible reason for founders’ persistence could be flow and team flow experiences. It is already known from other work contexts that flow is conducive to work satisfaction [4] and team performance [5]. In the field of start-ups, however, these relations are still largely unexplored. Therefore, flow as well as its antecedents and consequences are worth examining, especially in the early start-up phase, in order to benefit from its positive effects. Thus, the present paper aims to explore how founders get into flow and team flow and what impact this has on them and their work in the start-up process.

### Start-up companies and entrepreneurial challenges

Working on a start-up is associated with new opportunities, but also with challenges [1, 6]. For example, start-up founders may face a lack of support [6], and dealing with crises can also be highly demanding [7]. In addition, start-ups have often not yet developed routines to master their everyday business and meet challenges [8]. Thus, entrepreneurs are particularly exposed to stressors such as uncertainty [2, 9]. Although working in a start-up can be highly satisfying and a current meta-analysis found entrepreneurs to perceive higher well-being than employees of other organizations [9], the negative effects of entrepreneurial challenges, for example on an increase of the founders’ negative emotions, cannot be neglected [10].

As flow experience could be a possible outcome of a positive form of dealing with stressful events by interpreting them as manageable challenges [11] this mechanism should also be looked at in the context of start-ups. Hereby, flow could be an approach to deal with challenges and create a more positive work environment. However, it is unclear how flow can arise in the conditions of start-ups and what factors have an impact there. Therefore, the present study aims to create a profound understanding of flow in start-ups and to identify possible approaches.

### Flow and team flow experience

Flow experience is defined as a state of complete absorption and self-forgetting while performing a task that is perceived as optimally demanding [12]. During flow, one step seems to automatically follow the next, with no need to think about how the activity should be performed. At the same time, the individual’s entire attention is focused on the task and their thoughts do not wander. Irrelevant distractions are suppressed and a sense of complete control of a fluid process with little effort is experienced while time seems to be accelerated [12, 13]. Csikszentmihalyi was the first to describe the experience of flow when he observed people pursuing a wide variety of activities—some of them involving high risks—even though they were not rewarded for doing so [12]. In his interviews, he concluded that people who perform different actions, such as dancing, rock climbing or playing chess, experience similar situations and describe their activities as absorbing and extrinsically rewarding. Various antecedents show positive effects in different contexts and can promote flow. Peifer and Wolters suggest a framework that assigns them to three spheres: the individual, the task- or job-related, and the organizational or social sphere [14].

The phenomenon of flow experience can also be observed on the team level. Team flow is defined as a shared flow of a group or team in a social situation [15]. It typically results from optimal team dynamics during an interdependent task [16]. Beyond that, a distinction can be made between co-active flow, with team members experiencing flow individually, and interactive flow, requiring direct interaction and communication [15].

### Flow experience in innovative work contexts

Flow can be experienced in a variety of contexts at work and leisure. However, it is experienced more frequently at work [17, 18] and has several positive consequences, which can be beneficial in the start-up process. The consequences of flow can also be classified into the three spheres individual, task- or job-related, and organizational or social consequences [14]. Flow at work is usually perceived as a positive state and is shown to have positive effects on a person’s performance as well as on well-being and teamwork factors [for an overview see 14]. Moreover, it can have positive effects on creative behavior [19, 20]. In a study with music students, for example, it was shown that the flow when composing a piece of music is related positively to the creativity of the resulting composition [21]. Flow experience also has a beneficial effect on innovative behavior, as a study on consumer participation in product innovation showed [22].

Thus, it is likely that the flow experience also plays a role in the start-up process, which is characterized by a need for innovation [23]. Start-up founders are confronted with various challenges during the process of developing and implementing ideas [6]. At the same time, working on one’s own start-up is an activity characterized by intrinsic motivation [24] in which potentially flow-promoting conditions can be found. For example, according to a study on team leaders from different companies, flow occurs particularly in tasks such as planning processes, problem solving, and evaluation [25]—activities that could also be crucial in the start-up process. Furthermore, a study with students in a business simulation shows that flow has a positive effect on learning performance and this in turn on entrepreneurial self-efficacy [26]. The autonomy perceived and chosen by the founders also plays a pivotal role in the start-up [27]. At the same time, it is part of the Job Characteristic Model by Hackman and Oldham [28] and, according to the model, affects the perceived responsibility for work results and, as a consequence, the positive perception of these. The relationship between perceived autonomy and flow has already been observed in other contexts and could be of particular importance in start-ups [29].

### Research gap and aims of the study

Research to date shows controversial results regarding start-up founders’ experiences and well-being [9]. On the one hand, entrepreneurs are motivated by various factors to pursue their start-up [24] and show high levels of well-being [9]. On the other hand, opposite results, such as negative affective states triggered by different factors [10] and the confrontation with a variety of stressors in the start-up, are also evident [2]. Furthermore, a recent study showed that the stressors in the start-up context do not only have negative effects, but that the high demands are also associated with entrepreneurs’ well-being [30]. Here, the experience of flow could have played a central role. The challenges might have been just so difficult that the founders interpreted them as feasible and expressed exactly the right skills to overcome them. This could have led to a positive experience of flow as proposed by Peifer and Tan [11], which in turn could have had a positive effect on well-being. However, it is not yet understood how founders experience flow and what effects the experience has in the context of start-ups. Since many different factors can have an effect on the experience of flow and flow in turn can lead to a variety of consequences [14], a thorough analysis should be carried out here.

Flow and its consequences have been studied mainly using quantitative methods, but have not yet been considered in detail with reference to the start-up process, which may differ considerably from other work contexts regarding the challenges, stressors and motivational factors that can be experienced [6, 24]. In order to understand start-up founders’ experiences and support them in their work in the best possible way, it is necessary to identify which of the already known factors [14] and which additional factors are related to their flow and team flow. As Swann already stated, qualitative research approaches can provide detailed insight into how flow is experienced and can enhance the understanding of this state [31]. To identify so far unexplored aspects and beneficial factors, the qualitative approach shows great potential.

**Fig 1:**
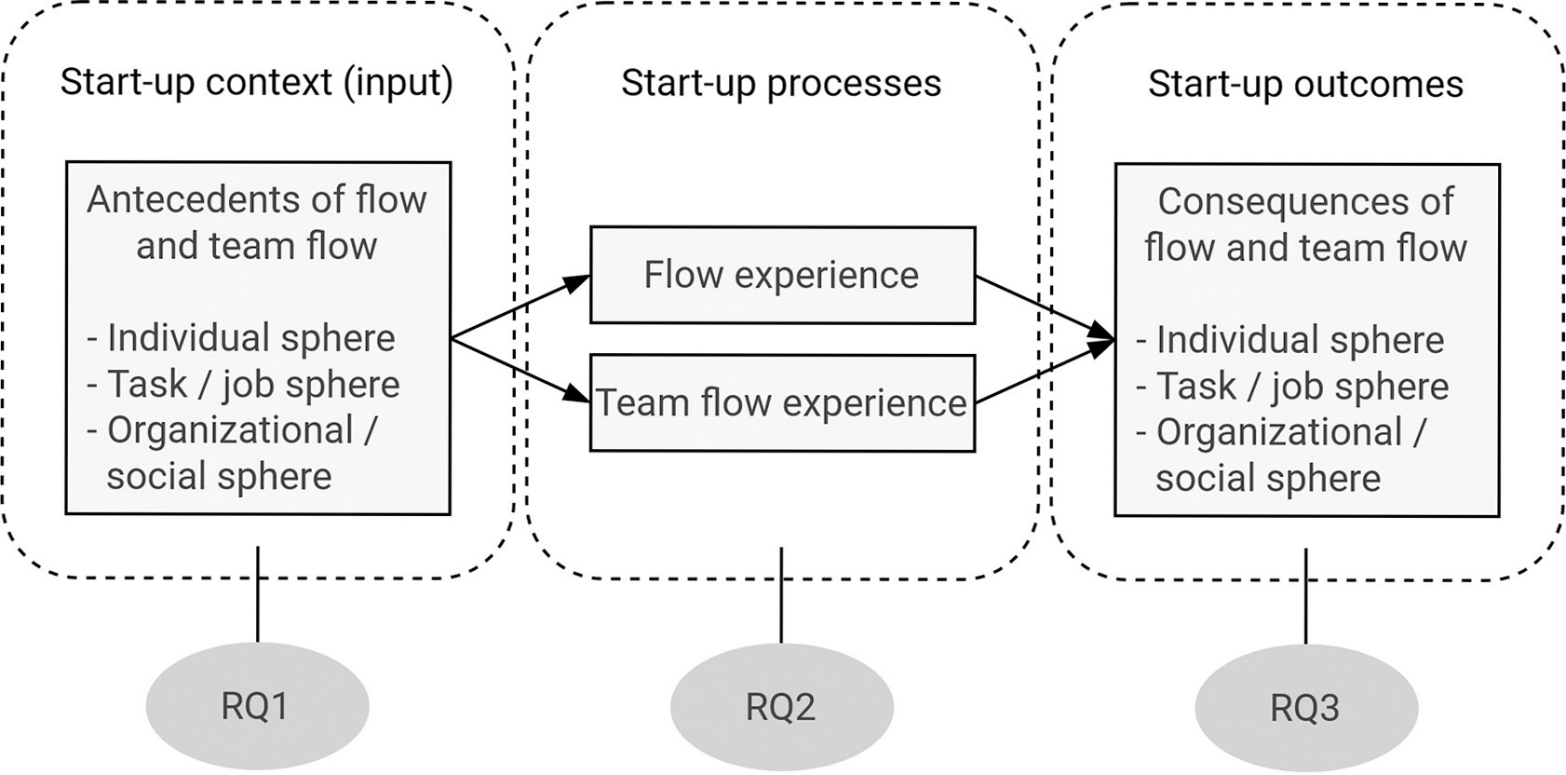
Derivation of the research questions from the relationship between start-up factors and flow and team flow in an input-process-outcome model.

The research questions (RQ) concerning start-up founders’ flow to be investigated through the interviews as illustrated in Fig 1 in an input-process-outcome model of the start-up context were the following:

1. RQ1: Which factors do founders experience as enhancing or inhibiting their individual flow and team flow?
2. RQ2: How do founders perceive flow and team flow and in which situations in the start-up work do they experience them?
3. RQ3: What are the consequences of flow and team flow in the start-up context and what role do these states play when working on the start-up?

## Method

In order to better understand the founders’ experiences, the qualitative method of a semi-structured interview-based study provides a suitable approach. It allows for an explorative analysis of the still rarely examined factors and effects of flow experience in the start-up process. Furthermore, the participants in their unique state during the founding process are given the opportunity to discuss their experiences in detail, which would be limited in a standardized questionnaire. Ethical approval for the study was obtained from the Ethics Committee of the University of Lübeck, Germany (21–465, 05/01/2022).

### Participants and procedure

**Fig 2:**
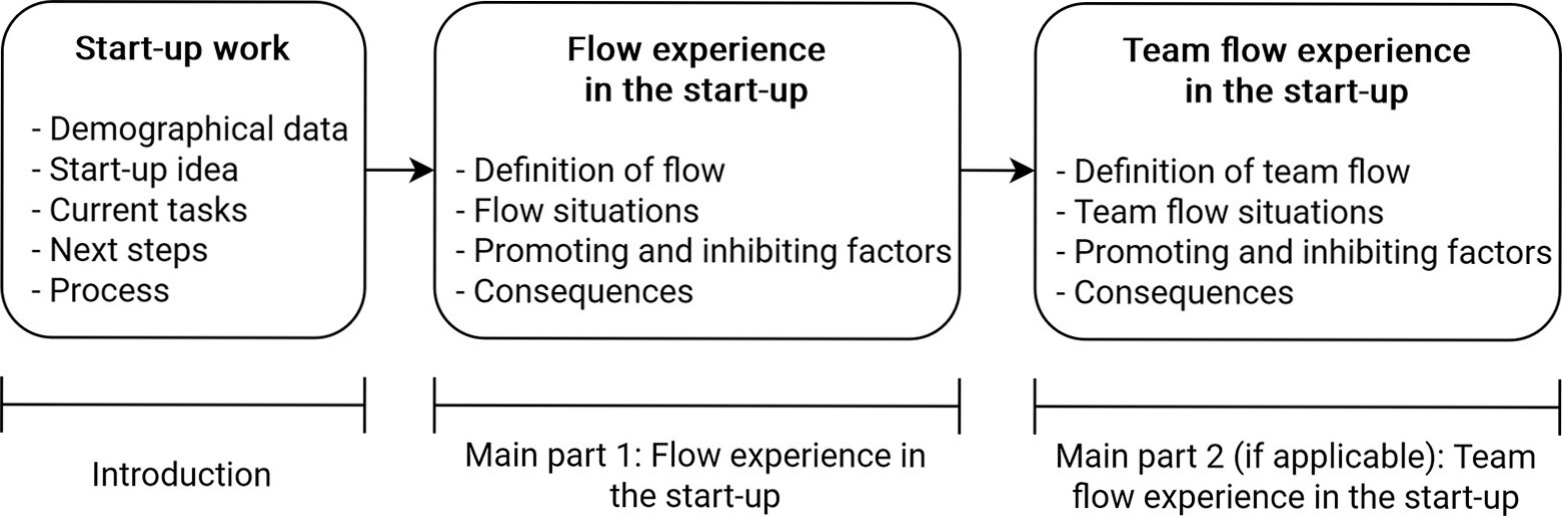
The interview guideline.

The participants in the interview study were 21 start-up founders or prospective start-up founders in Germany, Austria and Switzerland currently working on their own business ideas and about to formally establish a business or having done so within the past three years. The average age was 31; nine participants identified as female and 12 as male. They were recruited by convenience sampling via social media and were not rewarded for their participation. The interviews were semi-structured with an interview guideline, with questions adapted to the participants’ experiences aiming to establish an interactive conversational setting [32]. First, a pilot interview was conducted to check the interview guideline, and was not included in the analysis. The main topics of the interview guideline as depicted in Fig 2 were the participants’ individual flow and team flow experiences, focusing on typical situations of the experience, its antecedents, and consequences. Thus, the applicability of the already known characteristics of flow [13, 33] were to be examined for the start-up context. The factors associated with flow and team flow were to be identified in the individual, task-related, and organizational/social spheres, following the framework by Peifer and Wolters [14]. The complete interview guideline can be found in S1 File.

Prior to the start of the interview, all participants were given detailed information about the procedure and data protection and gave their written consent to the audio recording. The interviews, which lasted on average for 25 minutes, were conducted in German between January and April 2022 in digital form via video call using the video conferencing tool Webex. An audio file was recorded and subsequently transcribed verbatim.

### Qualitative content analysis

To structure and contextualize the participants’ statements, a content-structuring qualitative content analysis according to Kuckartz [34] was applied following the process steps in a computer-assisted form with the help of the tool MAXQDA. In the coding process, deductive and inductive codings were developed and applied to the transcripts. The deductive codings were based on the established characteristics of flow as described by Csikszentmihalyi [12] and on a framework developed by Peifer and Wolters [14], which assigns antecedents and consequences of flow to three spheres: the individual sphere, the job and task related sphere, and the organizational and social sphere. The inductive codings originated from the participants’ responses and complemented the coding frame that covered in detail the founders’ experiences and ideas regarding flow and team flow. Within the scope of self-reflective subjectivity according to Steinke [35], the interviews were coded by two members of the research team using the coding frame developed for the material. Subsequently, agreements and disagreements on codings were discussed in detail. The complete codebook can be found in S1 Table.

## Results

The content analysis allowed for the central aspects of flow and team flow to be identified. They are presented in the following in order of the topics addressed in the interview.

### Flow experience

First, participants were asked how they defined flow experience. Most participants were familiar with flow. As characteristics of flow they mentioned a strong focus of attention as well as fun while working on the task at hand.

*P6*: *When you have so much fun or focus on your work that you hardly even realize you’re working […]*

Participants also named a demand-skill balance in which the activity is neither too challenging nor boring. Other characteristics of flow experience the participants identified were a sense of autonomy, clear goals, motivation, and the feeling of fulfillment, while at the same time the sense of time is distorted.

*P7*: *I believe that you are happy at that moment and that time simply passes without your noticing it*.
*P5*: *And maybe also when I know that it is a linear way of working*. *What I do is that I have a clear goal in mind and can then also totally focus on it and achieve it very ambitiously at work in this situation*.

In addition, they reported a feeling of progress and increased productivity as characteristics of flow, as well as physical changes, such as forgetting about feeling cold or hungry. A few participants were not familiar with the term flow experience. Nevertheless, after flow was defined by the interviewer, they recognized the state and reported having experienced it.

### Flow situations

Next, participants were asked about typical flow situations in their everyday start-up activities. They named different types of tasks: strategic tasks such as business plan development, creative tasks such as designing, and tasks directly related to product development. They moreover mentioned systematic tasks such as accounting or technical as well as practical tasks.

*P9*: *Then I’ve definitely had that a couple of times now as well*, *in general when it comes to strategic planning*.
*P4*: *I think what definitely comes up a lot is when I have to think about things intensely and sort of let myself be creative […]*

Some could not describe a specific flow task and reported that for them there were rather different factors or characteristics of the task that can promote flow. For example, they described that they experienced flow primarily in tasks that coincided with their interests, related to learning experiences, or involved interaction in a team or with customers.

*P8*: *Well*, *I would say all the things which I have much fun with*. *That makes it easier to stay in the flow*.

In addition, flow was described as occurring more frequently in the early stages of the start-up. Some participants, however, reported having experienced flow more often in other areas of life than in start-up activities, explaining this with the turbulent and unpredictable work routine in start-ups.

### Factors conducive to flow

Next, participants were asked about factors having a positive effect on their flow experience. In the individual sphere, participants defined personal well-being as crucial for the flow experience in the start-up process.

*P3*: *And I have that very often when I feel well mentally […]*

In the task sphere they named the variety of different tasks and skills demanded, as well as the demand-skill balance of a task described as conducive to flow.

*P14*: *And from the task itself*, *I see in any case a certain variety*. *In other words*, *phases where it becomes more difficult*. *Phases where you can just rest a little bit mentally and put something together or so*, *and have a certain variety in any case*.

Moreover, (positive) feedback from others or from the task itself and autonomy or perceived control when performing a task are mentioned. In addition, participants explained that flow occurred especially when the task was perceived to be meaningful for the start-up.

*P3*: *[…] when I realized*, *oh*, *that’s something where I really pursue my mission in life*. *So*, *with this I fulfill a little bit of what I want to change in the world*.

The founders also described that clear goals and moderate stress could promote flow and that tasks offering a learning opportunity for the founder were more likely to be experienced in flow.

*P2*: *And the other thing is kind of a light pressure*, *I would say*. *So*, *not the deadline due in two hours and you’re screwed if you don’t make it by then*, *but so that it doesn’t hurt yet*, *it’s still okay just knowing you have to do it*.

On the organizational and social sphere, the work environment and the equipment of the workplace were mentioned as conducive to flow. Participants reported that a quiet workplace with no interruptions was also crucial to a flow experience. They explained that, depending on personal preference and the nature of the task, both individual work and interaction with the team could promote flow.

*P11*: *And environment*, *for me a room where I feel comfortable […] where I can create the atmosphere*.
*P21*: *And I think*, *in general*, *a healthy balance between communication and also being able to work alone and keep going*.

Also, as a factor at the interface between the social and the task sphere, several participants reported that personal interest in performing a task influenced their flow, resulting in more flow when the characteristics of a task matched a person’s strengths.

### Factors inhibiting flow

The participants were asked not only about factors that enhanced their flow, but also about those that inhibited it. Again, the factors named can be assigned to the spheres of the framework.

In the individual sphere, participants identified physical limitations, e.g., physical pain, preventing them from performing the flow activity. In the task sphere, they named being overwhelmed by the task as a crucial factor. A disruption of the demand-skill balance led to a lack of flow. In this context, failures in present or previous tasks and a lack of personal interest in a task were named as inhibiting factors.

*P5*: *[…] when I’m overloaded*. *That is*, *when I’m stuck on something and don’t know how to continue*.

Multitasking or a heavy workload in other fields, for example due to a second job in parallel to the start-up, also inhibited flow in start-up related tasks and can also be assigned to the task-related sphere.

*P2*: *Yes*, *I think the typical thing is that you have something else to do that is more important or at least has a high priority*, *so that you can’t fully focus on it*. *So*, *for quite a while I was still working in the hospital at the same time when founding the start-up*, *and it’s obvious that when you’re working there*, *you’re not able to focus on your goal*, *“flowing” in your start-up*.

In the organizational and social sphere, many participants described distraction as an inhibiting factor of flow. In addition, they named dependence, for example on the results of others, and thus limited autonomy, as an obstacle to flow. Within the start-up, online communication and meetings were perceived as not beneficial, and conflicts—both within the start-up team and in the private sphere—had a negative influence on flow experience.

*P19*: *[…] disagreements in the team—you also have that sometimes—this disrupts the flow*.

A factor related to all three spheres was stress in terms of a lack of time or high performance demands. The reasons for the stress or pressure the founders felt could be caused by themselves, such as high expectations regarding their own performance or due to external factors like challenging start-up stages with lots of new situations to be mastered.

*P10*: *So somehow pressure factors*, *so that I think I have to perform now*. *Or I have to finish this and that by the end of the day*.

**Fig 3:**
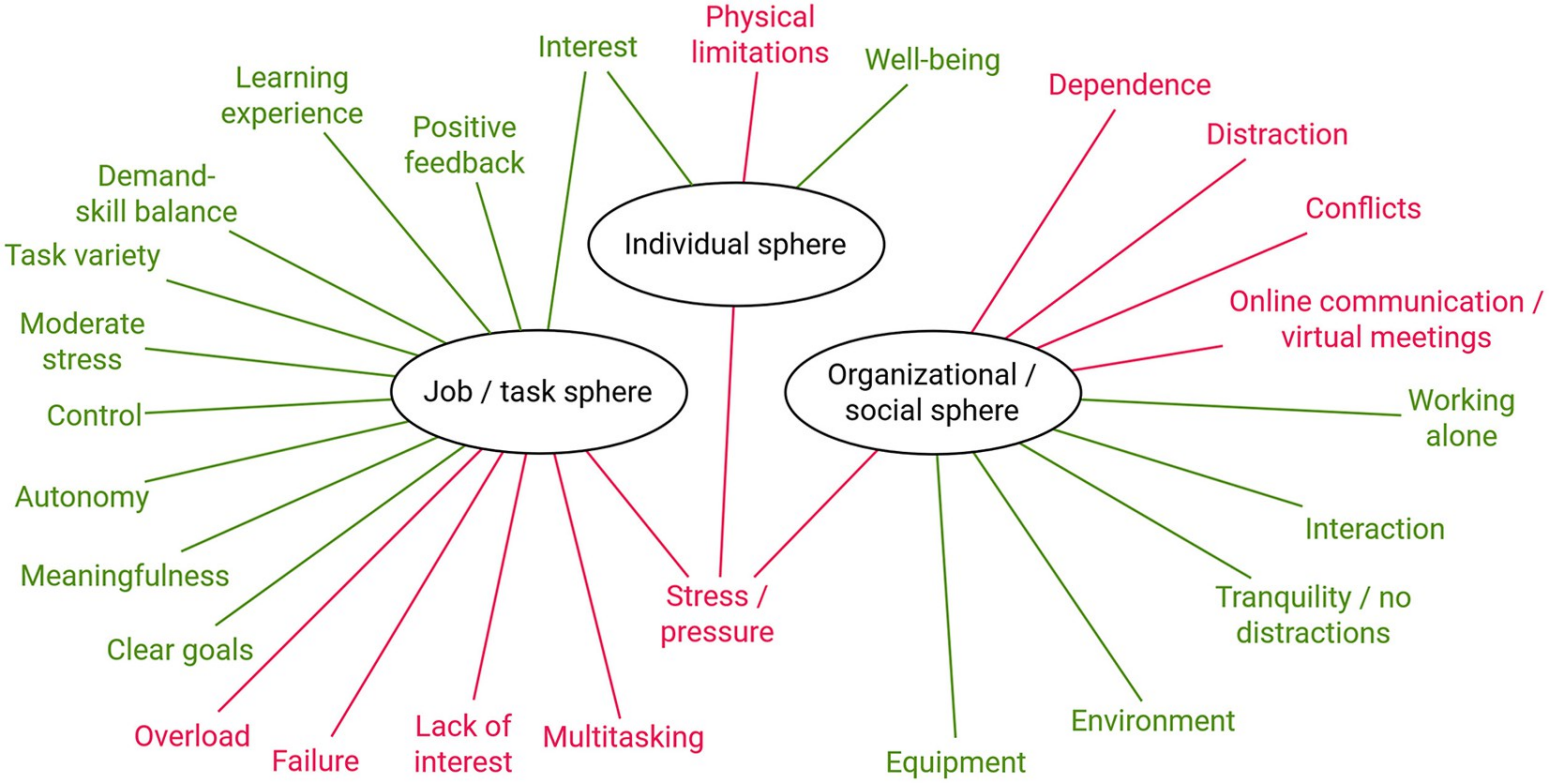
Start-up factors enhancing flow (green) and inhibiting flow (red).

Fig 3 shows all factors conducive to and inhibiting flow that were mentioned by the participants.

### Consequences of flow

Next, we asked the participants about the consequences of their flow experience in the start-up concerning the individual, task- and job-related as well as organizational and social sphere. The participants recognized various consequences of flow in the personal sphere. They reported more fun at work and increased motivation. Moreover, they attributed the feeling of being full of energy and satisfied with their own work to their flow at work. Moreover, according to the participants, flow can have a positive effect on resilience and self-efficacy.

*P20*: *I somehow just get more accomplished*, *I’m more convinced of myself that I can do things*, *I just feel a lot of joy*.

In the task and job sphere, better progress at work as well as better results in work tasks were mentioned. In addition, participants reported that they worked more in flow and also mastered unpleasant tasks, stating that flow helped them to make progress during challenges or periods of stagnation. They also mentioned learning experiences and the development of new ideas due to flow.

*P10*: *Yes*, *it was just extremely productive*, *extremely good*. *So effective*, *efficient*. *Just in terms of the use of time*, *that I simply managed to get a lot done in a short period of time*, *I was incredibly productive*. *And that it was just a great result*.

Likewise, the effects of flow on teamwork were reported and could be assigned to the social and organizational sphere. For example, participants described being able to engage more with others in flow or to experience improved collaboration.

Apart from the positive effects of flow, a few participants also mentioned some negative effects, namely that in flow one sometimes tends towards perfectionism and might become obsessed with details, even if unnecessary. This can be assigned to the individual sphere. As a negative consequence in the job sphere, it was also stated that flow in the start-up process might lead to other tasks and obligations being neglected.

*P19*: *[…] because you do a lot in a very short time*, *because you only think about the one thing and maybe neglect other things*. *But still*, *the experience is very positive*.

In the organizational and social sphere participants described a lack of team communication due to individual flow leading, for example, to decisions being made hastily.

*P19*: *Yes*, *also because in my opinion flow also leads to the fact that one rushes ahead a little bit and makes hasty decisions*, *because you are in flow and then you just call a few people or settle things that are perhaps not planned or discussed in detail yet*.

As a consequence, it was also mentioned that in flow the focus of attention was narrowed, which could be either positive or negative. Depending on the task, this might be helpful or could impede openness to new inspirations while the attention was overwhelmingly on details.

*P4*: *When you’re in the flow*, *it’s just relaxed and you’re so extremely focused*. *And I think that sometimes it’s also good to be torn out of this pure focus*, *because then it can of course also happen that you miss some things*.

By contrast, it was also pointed out that in some situations and tasks flow had little to no effect and was not necessary, for example because the outcome of a task is not variable and cannot improve in flow.

**Fig 4:**
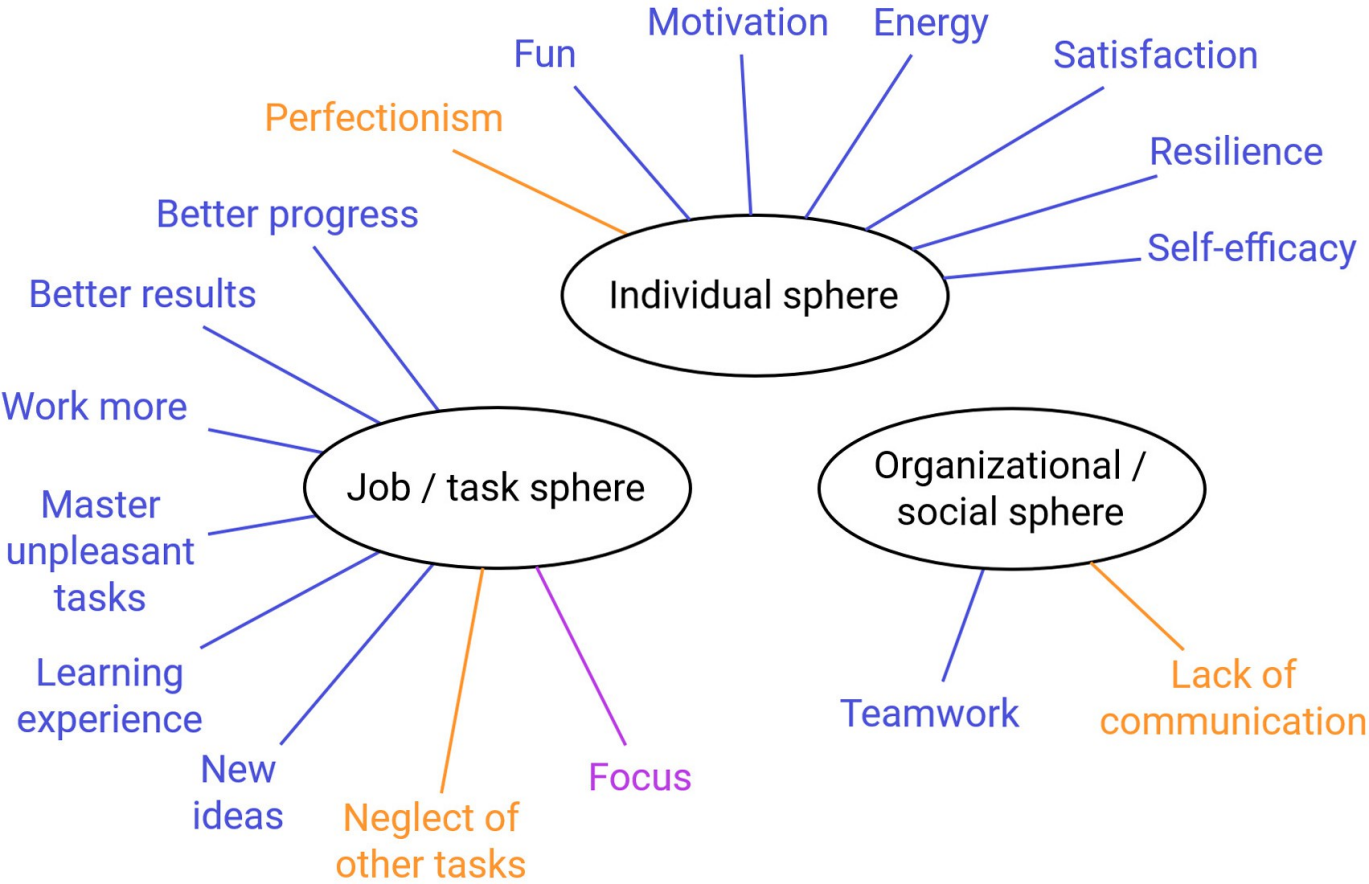
Reported positive (blue), negative (orange), and nondirectional (purple) consequences of flow.

Fig 4 shows all consequences of flow in the start-up that were mentioned by the participants.

### Team flow experience

In the second part of the interview, we discussed the phenomenon of team flow with participants collaborating with a team on the start-up. As with individual flow, we first asked them to define team flow. In general, it was confirmed that flow could be experienced in a team. As central characteristics, the participants mentioned that in team flow the team members must engage with each other and that, unlike in individual flow, interaction was common.

*P6*: *Yes*, *definitely just by interacting*. *So*, *the individual flow is really that you kind of sink into your figures and can block out everything around you*. *Of course*, *it’s a different situation in the team because you have an incredible amount of interaction and it’s a different way of working*, *a different feeling*.

Similar to individual flow, clear goals and focusing on the task at hand were mentioned as characteristics. The participants moreover reported that team flow could also occur as a combination of individual flow experiences and that individual flow could stimulate other team members’ flow, thereby creating team flow.

*P10*: *So I had the feeling that in the team it’s mainly about one idea sparking the other*.

### Team flow situations

The typical situations in which team flow was experienced frequently coincided in many parts with those of individual flow. Thus, team flow was also reported to be experienced in creative, strategic, and product development tasks, and generally tasks that matched personal interests.

*P6*: *Product selection*, *definitely*. *So it’s super exciting when you can really imagine what the product looks like and then finally have the product in your hand at some point*. *That’s very cool and fun*, *because it’s really about what you want to do in the end*.

However, participants most frequently mentioned generally experiencing team flow in collaborative tasks or in team interactions. New tasks were also described as suitable for team flow.

*P19*: *And that’s how it was that we always communicated with each other throughout the day*. *Person A does this*, *person B does that*, *persons C and D do that together*. *That was a very fulfilling experience or a good feeling […]*

### Factors conducive to team flow

Also regarding team flow, participants were asked about conducive factors. Again, it was found that many different antecedents in the individual, task-related, and organizational and social sphere influence team flow in the start-up. The individual sphere included factors conducive to team flow concerning the individuals in the team. As for individual flow, the participants named well-being as a crucial factor.

In the task sphere, similar to the results for individual flow, clear goals and moderate stress were named as being conducive to team flow.

*P19*: *Then this*, *I’ll call it positive stress before the launch*, *as I said*, *is definitely something that has brought us forward*.

In addition, participants reported that the sense of autonomy, positive feedback, and perceived meaningfulness of the task could facilitate team flow.

In the organizational and social sphere, an additional factor regarding team flow was personal contact. The founders experienced team flow more frequently in face-to-face interaction than during virtual teamwork. Furthermore, they described their work environment and working without interruptions as crucial factors for team flow, as they had done for individual flow.

*P10*: *And then it was also the environment*. *I’m very sure that it’s just because of it*. *[…] [In the co-working space] they have different rooms for co-working or for seminars or something like that*, *and the rooms are especially designed to stimulate creativity*, *and that really works*.

Furthermore, as a factor at the interface between the individual and the task sphere, the participants stated that team flow depended on the team members’ interests and if these coincided with the characteristics of a task, explaining that tasks that are fun for the team members are more conducive to team flow.

*P2*: *[…] it just depends very much on how enjoyable the tasks are or not*.

As factors combining the individual and social sphere, they also stated that there must be a common basis in the team or team members have to be prepared individually for a certain task to experience team flow together, for example, referring to matching prior knowledge and skills.

*P5*: *Then*, *that the knowledge base is the same*. *In other words*, *you don’t have to inform everyone about the current situation*, *everyone is on the same level*.

Similarly, they described the team members’ commitment as beneficial, i.e., identification with common goals, and a general team spirit in the form of trust and mutual support.

*P13*: *So I think that the relationship between people also helps a lot and is important*. *I couldn’t imagine working so much and so closely with someone I didn’t somehow like*. *Even if the person has super-good skills*, *I mean*.

### Factors inhibiting team flow

Similarly, the factors inhibiting team flow coincided in many aspects with those for flow. In the individual sphere, the participants mentioned physical factors such as physical pain as a hindrance to team flow, as in the case of individual flow. In the task sphere, they also named overload as an inhibiting factor. However, the daily routine was also described as an obstacle; a lack of challenges inhibited team flow. Participants similarly described failure in previous tasks or the lack of feedback in terms of a missing visibility of the results of a task as unfavorable for team flow.

*P3*: *Sometimes*, *when you’re in the daily routine or you’re not making good progress on something*, *on an existing issue*, *then I notice that it leads to fewer experiences like this*.

A further obstacle in the task sphere was multitasking, as start-up founders most times have parallel jobs with different demands to handle, especially in early start-up stages.

*P6*: *We also have the problem*, *of course*, *that not everyone always has time*. *Two of our co-founders also have full-time jobs*. *So it’s difficult*, *let’s say*, *to take the whole day*. *But when we do*, *it always has a positive effect on us*.

In the social and organizational sphere, dependence on external factors and interruptions were mentioned to negatively influence team flow. In addition, the participants explained that online communication was often not suitable for team flow and represented a further barrier. They also stated that any kind of conflict in the team or in the team members’ private lives could negatively affect team flow.

*P19*: *Well*, *disagreements*, *as well*. *Half a year ago*, *there was a really critical situation*, *because we had disagreements in the team*. *That definitely killed the flow for a few weeks*.

As a factor related to all spheres, the founders mentioned stress as inhibiting flow. Stressed team members, whether due to high demands in the start-up or external factors, did not experience flow together. In addition, it was pointed out that a lack of a common basis could be a problem. This factor can be assigned to the individual and the social sphere. There was no shared flow if, for example, the team members had different levels of enthusiasm for the start-up idea.

*P11*: *When one is more convinced than the other*. *Let’s say when the common mission is disrupted*.

**Fig 5:**
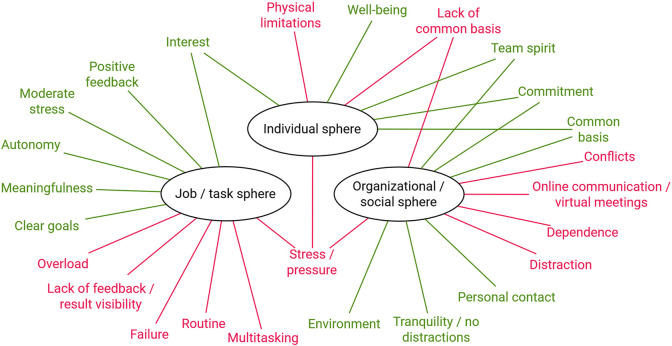
Start-up factors enhancing team flow (green) and inhibiting team flow (red).

All factors mentioned to be conducive to or inhibiting team flow are presented in Fig 5.

### Consequences of team flow

Concerning the individual sphere of team flow consequences, the participants described increased satisfaction and motivation, both when facing new challenges and while moving forward in stages of normal routine. In the task and job sphere, many participants emphasized making better progress and getting improved results in team flow, just as with individual flow.

*P6*: *Being focused and in the flow together*, *then you can achieve quite a lot*.

Beyond that, the main consequences of team flow described were those affecting the team itself and thus attributable to the organizational and social sphere. The participants reported that experiencing team flow together had a positive effect on their team spirit, thus promoting trust among the team members, and allowing them to grow together as a unit. In this context, they also mentioned increased collective efficacy, similar to self-efficacy in individual flow.

*P9*: *Well*, *at that moment*, *it feels like we’re getting closer again in the team*, *I’d say*, *or more closely connected*, *that’s what I would call it now*, *from a team perspective*. *And that you just feel more like a unit*, *or this team spirit that we shape and are the company*.

A negative consequence of team flow was also mentioned and could be assigned to the individual sphere. A participant noted that team flow caused the team to be more vulnerable to setbacks, meaning more difficulty dealing with periods of less progress after the positive experience of team flow had been shared as a team.

*P17*: *However*, *if you’re always in this flow mode*, *things are going very well*, *you’re making progress*, *then you can also become dissatisfied quite quickly if small things now set you back again*, *for example*.

As for individual flow, participants reported a strong focus during team flow that could both help or hinder, representing an undirected consequence of team flow that, depending on the task, can be seen as positive or negative.

**Fig 6:**
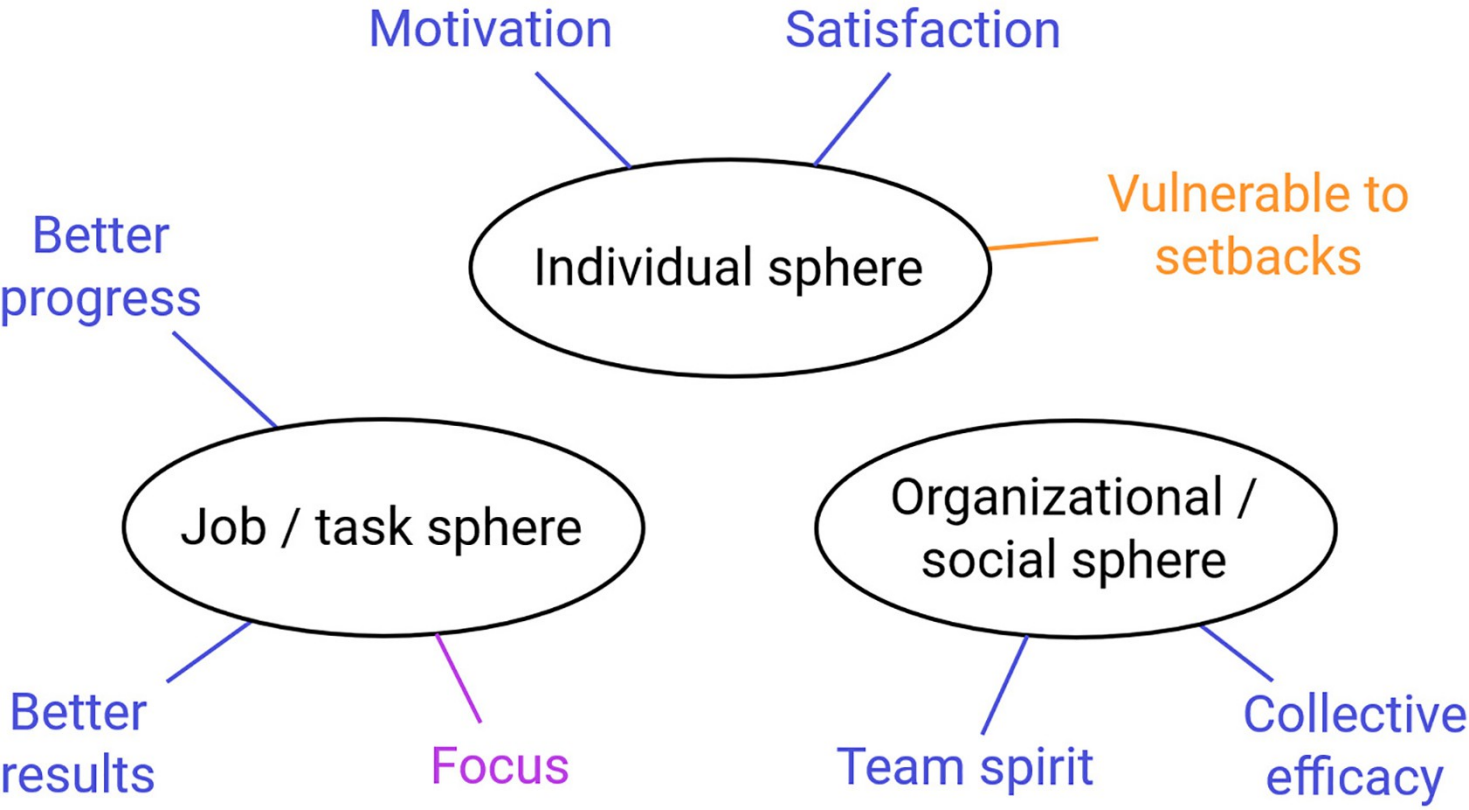
Reported positive (blue), negative (orange) and nondirectional (purple) consequences of team flow.

The consequences of team flow that were mentioned by the participants are summarized in Fig 6.

## Discussion

The present study was intended to shed light on the role of flow and team flow experience in start-ups. In semi-structured interviews, founders explained their unique work situations when creating a new venture. They reported on typical flow situations an also on promoting and inhibiting factors. In addition, the consequences of flow and team flow for their work outcomes and the start-up teams were identified.

### Flow and team flow experience

The majority of the participants were familiar with flow as a psychological state and were able to name specific situations and also promoting and inhibiting factors. This indicates that start-up founders are a very well-informed and self-reflective group, which explains the broad outcomes of the interviews.

The participants’ descriptions of flow are consistent with the definitions in established models [12, 33]. The reports suggest that the participants were familiar with working in flow and that a start-up offers great potential for flow situations. The start-up founders experienced flow, for example, in creative tasks, strategic tasks, and product development. This is in line with prior research showing that flow plays a role especially in creative and innovative work processes, in which various antecedents favoring flow can be found. For example, the method of design thinking can promote flow, as a study by Yang and Hsu showed [36]. In addition, it was confirmed that flow is experienced in activities like planning, problem solving, and evaluation tasks [25].

Team flow was also familiar to most participants working on their start-ups in teams. Situations of shared flow seemed to occur repeatedly in the start-up context. On the one hand, similar contexts were mentioned as for individual flow, such as creative tasks and strategic tasks. On the other hand, the emphasis was clearly on the interaction in the team and collaborative tasks were described as team flow situations. This is in line with conceptions suggesting that interdependent tasks are a typical context for team flow to occur [15, 16, 37].

### Factors conducive to flow and team flow

Regarding flow and team flow promoting factors, many aspects were named that have already been described in the literature to have positive effects on flow. In the task sphere there are several flow promoting factors the founders experienced that are described by Csikszentmihalyi as being among the main elements present when experiencing flow, for example perceived control and the challenge-skill balance [38]. Also, tasks with clear goals [39] as well as learning situations [40] as mentioned by the founders are described as typical flow contexts. In addition, the founders name factors such as autonomy, feedback, variety and meaningfulness as promoting factors that can be assigned to the task sphere. These factors can also be found in the Job Characteristics Model [28]. Research has already confirmed the positive effect of these factors on flow [4, 41].

The slight pressure that fosters flow and team flow as described by the founders is also consistent with the results of earlier research on individual flow. For example, Peifer et al. suggest that there is an inverted u-shaped relationship between flow and arousal, which indicates a positive relationship between mild stress and flow [42].

In the organizational and social sphere, too, factors were identified that are already known from existing research. The general observation that both working alone and in teams can stimulate flow has been researched in other contexts [15, 43] and was confirmed here by the founders. Also, their perception of personal contact as being more conducive to team flow than remote teamwork is in line with suggestions from Peifer et al. and provides empirical evidence for it [37]. The quiet setting without interruptions that the founders perceived retrospectively as conducive to flow and team flow could be explained by the strong focus of attention during flow that makes it possible to exclude external events [44]. A novel aspect of the present study is the extension of these findings to team situations and to the start-up context.

Other factors that seem to be particularly relevant in the start-up context are flow-promoting equipment and environment. For the latter, there is no specific flow-facilitating environment, but rather a person-environment fit: The founders reported that the workspace must meet their needs and the demands of the task in order to be conducive to flow. Thus, for creative tasks, a different environment could promote flow than that for strategic tasks. This is in line with findings from environmental psychology stating that different environments serve different needs [for an overview see 45] and applying them to flow research.

Interest in a task is another flow-promoting factor named by the founders that can be assigned to the interface between the individual and the task-related sphere. This has been reported in the literature not only as a predictor of flow, but also as a moderator of the positive relationship between the perceived challenge-skill balance and flow [46].

On the team level, the founders explained different team aspects that favor especially the shared flow experience, such as a general team spirit, a common basis and commitment, that can be assigned to the interface between the individual and social sphere. In a similar way, van den Hout et al. define collective ambition, high skill integration, and mutual commitment as prerequisites of team flow [16]. The common basis, i.e., balanced competencies and expectations, is found in the theorized preconditions of social flow by Walker describing that every group member knows the competencies of every other and even goes beyond this assumption [15].

### Factors inhibiting flow and team flow

Among the inhibiting factors for flow and team flow, factors known from earlier research as well as new factors in this context were mentioned. In the individual sphere, physical constraints were described as an example—an aspect that has received little attention in research to date. Moreover, in the task sphere, the mismatch between one’s own needs and the characteristics of the task may inhibit flow, for example in the form of a lack of new challenges or excessive demands that cannot be fulfilled. An imbalance between the challenges of the task and individual skills inhibits flow and team flow [12]. This is also in line with our result that perceived pressure to perform or limited time to complete a task could lead to a perceived overload and thus inhibit flow and team flow.

The negative effect of multitasking on flow as described by our participants has also been shown in earlier research [47]. Here, our study supports this finding using a qualitative approach. Also, it adds empirical evidence that the effect is not only valid for individual flow but also for team flow.

Start-up teams also seem to suffer from less successful work periods, as the participants described reduced flow and team flow due to failure in previous tasks or the lack of visibility of one’s own results. The visibility of the results and thus feedback from the task is known from the job characteristics model [28], which has already been shown to be related to flow [41]. Performance accomplishments are considered a source of self-efficacy [48], which in turn is positively related to flow [49]. Failure in tasks within the start-up could reduce this self-efficacy and collective efficacy at team level and thus inhibit flow.

Regarding interdependent tasks as a factor in the organizational sphere, the founders described both positive and negative effects. On the one hand, team flow was experienced primarily in collaborative tasks. On the other hand, dependence on others was mentioned as a potential impeding factor for flow and team flow—particularly when the team members’ expectations and motivation did not match. This is in line with the findings of Aust et al., which show that a lack of shared mental models, i.e., shared assumptions about expectations and procedures within the team, can have a negative impact on team flow [50]. Similarly, participants reported that conflicts in the team may inhibit flow and team flow.

As an impeding factor at the interface between the individual and the task sphere, a lack of interest was mentioned. In order to achieve flow, a match between one’s own interests and the task is beneficial, which at the same time should not be too demanding but match the individual’s own skills [46].

### Consequences of flow and team flow in the start-up

In the individual sphere, the positive effect of flow and team flow on work satisfaction described by the participants is already known for flow from earlier research [4]—a novel aspect of the present study is the extension to the team flow experience. Moreover, participants reported increased motivation, energy, and fun as consequences. This concurs with findings suggesting a connection between flow and fun at work [51]. Furthermore, flow is already known to result in a feeling of greater vigor or sense of energy [52]. Also, the phenomenon of intrinsic motivation during the flow experience has already been described [53]. The perceived higher self-efficacy—collective efficacy at the team level—described by the participants is in line with findings of Salanova et al. confirming the positive relationship between flow at work and self-efficacy which becomes apparent in the form of an upward spiral [49]. In addition, the resilience reported as a consequence of flow in the start-up has recently been suggested as an outcome of the flow experience that serves as a mechanism to deal with stressful events at work in a positive way [54]. Furthermore, it has been examined in terms of positive correlations between flow and psychological capital, a concept including resilience as one of four positive psychological factors [55].

In the task sphere, the founders described both better results and better progress as consequences of flow and team flow. The positive effect of flow on performance measures has been described in earlier research [56], likewise the effect of team flow on performance [57]. The new ideas resulting from flow as explained by the participants have also already been reported in earlier research, for example, in terms of a positive relationship between daily flow and daily creative performance [20]. Moreover, the reported improved mastery of unpleasant tasks in flow is in line with earlier findings showing less procrastination in a learning setting when experiencing flow [58]. The founders’ statement that they worked more as a result of flow can be explained by the already known association between engagement and flow [59]. Regarding the learning experience participants report, Csikszentmihalyi describes growth and the development of new skills as a result of flow, which in subsequent flow activities leads to the search for new challenges—through which further learning takes place—in order to maintain a challenge-skill balance [60].

Regarding the team, the participants mentioned positive effects of flow on team processes like commitment or an improved team spirit in general. This fits with findings by Aubé et al. showing a positive effect of flow on team goal commitment that consequently enhances team performance [5]. In line with this, van den Hout et al. [57] describes a positive effect of team flow on team positivity.

Besides the mainly positive consequences of flow and team flow, some participants also mentioned potentially negative consequences—among these perfectionism. A possible explanation for this may be the high intrinsic entrepreneurial motivation among founders [24], which then coincides with the strong focus in flow [12] and the autonomy in the start-up context [27] potentially resulting in founders losing themselves while working passionately. Another negative consequence described was being more vulnerable to setbacks. This could be explained by the improved performance resulting from flow [56], in which founders may develop even higher expectations regarding their own work. These then can no longer be met in phases without flow. In the task sphere a negative consequence that was mentioned is neglecting other tasks. Csikszentmihalyi already claimed that nothing else seems to matter during flow, as the focus is so intense that individuals forget about other things [60].

Regarding the consequences reportedly positive and negative, the strong focus during flow is known as a typical characteristic of this state [12]. In the present study, it is emphasized that this aspect of flow may have a decisive influence on the results of a task done during flow. The strong focus of attention influences the perception and way of working during flow and team flow. The effect of flow on decision-making described by the participants could be explained by the increased self-efficacy associated with flow [49]. The founders could feel more confident to manage decisions on their own, which may be positive or negative, depending on the context.

Overall, however, flow and team flow seem to have mainly positive effects for individuals and teams in start-ups. Therefore, it is recommendable to support flow and team flow among founders.

### Practical implications

Many participants describe being subjected to great pressure and also having high expectations of themselves and their start-up. At the same time, they describe mainly positive effects of flow and team flow in the start-up, for example, in terms of increased personal motivation, satisfaction, performance or better team processes. Therefore, it is recommended to systematically promote flow and team flow in the start-up context and to help founders benefit from the positive consequences of these states.

Based on our findings, we propose applying approaches to promote flow and team flow in the start-up in the individual sphere as well as in the task-related and organizational or social spheres. In the individual sphere, it is suggested to allocate tasks in the start-up according to interests, so that flow can be generated during tasks matching the individual preferences.

To create more flow-promoting work taking account of the factors identified in the task sphere, founders should try to avoid overload and multitasking. Even with the high workload in a start-up, it is not helpful to work on things in parallel as this may impede flow and team flow—states that influence progress and the results of the start-up work. In addition, in order to achieve a shared flow experience, care should be taken to ensure that team members formulate clear goals for their tasks and perceive meaningfulness in their work, which should preferably be in line with the goals of the start-up itself. Regular interaction and reflection within the team could be helpful in this regard.

In the organizational sphere a key factor many founders describe is the design of a flow-conducive environment. As our results show, personal preferences determine what such an environment can look like. For founders, it can be a first step to becoming aware of their own preferences and to select specific environments for certain tasks, thus, for example, alternating between home office, public spaces, and co-working rooms. For the providers of co-working rooms, it is also important to design a flexible and diversified environment offering different spaces for different needs.

A way to implement the approaches outlined could be to create workshops for start-ups. As flow and team flow are experienced differently between different individuals and teams, workshops should focus more on reflecting on one’s own flow situations and the factors that promote or inhibit flow, rather than providing general instructions on how to experience it. In a workshop, following the three spheres of the model [14] the start-up team can explore how to create flow-promoting work, how to benefit from the flow in the team and at the same time how to be aware of the possible unfavorable consequences.

### Strengths, limitations, and directions for future research

To the best of our knowledge, the present study is the first to focus on flow and team flow experience among founders and to assess this with the help of qualitative interviews. The present study was able to reproduce many already known promoting and inhibiting factors for flow and team flow and the corresponding consequences, confirming the structure of the three-sphere model as suggested by Peifer and Wolters [14]. Applying a qualitative approach, it was possible to show which factors have a particular effect in the context of start-ups and have been rarely considered in the research so far, among them team processes in newly created teams and factors in the working environment and equipment. In addition, we identified mostly positive and also some negative consequences of flow and team flow in start-up teams. For example, on the one hand positive effects on the motivation to pursue one’s own goals in the start-up were mentioned as a consequence of particular interest in the start-up context. On the other hand, negative consequences such as perfectionism and a lack of team communication were uncovered, which had previously received little attention in flow research.

To ensure rigor and trustworthiness of the present study, the quality criteria of qualitative research according to Steinke [61] were taken into account in the research process. Thus, self-reflective subjectivity was ensured by discussing the coding frame and its application during the process among the analyzing team. In sampling and conducting the interviews, the aim was to create a dependency-free setting in order to establish an open and unbiased atmosphere. Thus, no prior relationships existed between the researchers and the interviewees. In addition, effort was made to represent the perspectives and values of the participants as broadly as possible in order to meet the criterion of authenticity. However, an issue that was not considered in the present study is the concept of triangulation. By adding further data sources, future research should consider putting the statements into a broader context.

A qualitative approach offers the potential to uncover previously unknown issues. This can provide a starting point for future research questions regarding flow and team flow in start-ups. In this context, the results found in the present study should in future studies be quantified with larger samples. In addition, it could be interesting to match the experienced flow and team flow with the innovation outcomes of start-ups to find if the perceived better performance actually results in better company results. Also, the selection of participants in the study through convenience sampling in a future iterative research process should be replaced by successive selection.

## Conclusion

Working on one’s own start-up is challenging, but at the same time rewarding and meaningful for many founders. Flow and team flow can make an essential contribution to start-ups, helping them to progress on the one hand and not to lose motivation even in times of doubt on the other. Therefore, founders of start-ups should try to recognize and cultivate their personal flow. This study identified many flow-promoting and inhibiting factors, which can help to increase the frequency of founders’ future flow and team flow.

## Supporting information

S1 File Interview guideline. (PDF)

S1 Table Codebook. (PDF)
