# Supplementary material for: Founders’ flow: A qualitative study on the role of flow experience in early start-up stages
Source: PLoS One. 2023 Oct 5;18(10):e0292580. doi: 10.1371/journal.pone.0292580 (PMC10553822; doi:10.1371/journal.pone.0292580)
Supplement: S1 File — (PDF) [file pone.0292580.s001.pdf]

Supporting Information 1

Interview guideline

The interview guideline serves as an orientation for the course of the interview. It is not rigidly followed and read out, but flexibly adapted to the course of the dialogue and the natural dynamics of the conversation. This means that blocks of topics do not have to be asked in the prescribed order if the interviewees have already mentioned other aspects that suggest a transition to a particular block of questions. The exact wording of the questions does not have to correspond exactly to the wording used in the interview, but serves as a guideline and orientation.

Introduction

Thank you for your participation in this interview. As part of my PhD project, I am investigating the flow experience of aspiring founders. In doing so, I would like to talk about the stages and activities of the start-up process in which flow is experienced, which factors promote flow, and which effects flow can cause. First, however, a few questions about yourself:

- Please tell me your age, gender and career background.
- In the last few months, you have been working on your own start-up idea (and have already founded a company). Please briefly describe your project and your start-up idea. Which industry does your start-up belong to?
- Have you ever started a business before?
- What are you currently working on?
- At what point in the start-up process are you right now? What have you already done and what are your next steps?
- How would you describe the startup process overall?

Flow experience

- Does the term *flow experience* mean anything to you? How would you define it for yourself?
- We define flow experience as the experience of being completely absorbed in an optimally demanding activity. During flow, the entire attention is focused on the task and the thoughts do not wander. The impression of merging with the activity is experienced.

- Have you ever had this experience at work? (If no: How would you imagine such a situation?)
- Can you remember a situation in which you experienced flow? Try to imagine yourself in that situation again. How did it feel?

Factors promoting or inhibiting flow

- In which stages of the process of working on your idea have you experienced flow so far?
- Can you give examples of activities in which you experience flow?
- What characterizes these phases / activities?
- Are there factors that particularly support you experiencing flow in these phases / activities?
- What factors prevent or interrupt your flow?

Consequences of flow

- What effects does the flow experience have on your work process?
- How does the work in flow influence the progress in the start-up?
- What changes can you observe compared to other work phases?
- When is flow helpful for working on the start-up idea?
- Are there also phases when flow is not helpful for the start-up process?

Team flow experience

(Questions to ask if the start-up is a group project)

- Do you experience flow only alone or also in a team?
- How would you describe the shared flow experience?

Factors promoting or inhibiting team flow

- In which work phases have you experienced flow together so far?
- Can you give examples of activities in which you experience flow in the team?
- What characterizes these phases / activities?
- Are there factors that particularly support you experiencing flow as a team during these phases / activities?
- What factors prevent or interrupt team flow?

Consequences of team flow

- What effects does the team flow experience have on working together as a team?
- How does team flow influence progress in start-up?
- When is team flow helpful for working on the start-up idea?
- Are there also phases when team flow is not helpful for the start-up process?
