# Supplementary material for: Founders’ flow: A qualitative study on the role of flow experience in early start-up stages
Source: PLoS One. 2023 Oct 5;18(10):e0292580. doi: 10.1371/journal.pone.0292580 (PMC10553822; doi:10.1371/journal.pone.0292580)
Supplement: S1 Table — (PDF) [file pone.0292580.s002.pdf]

Supporting Information 2

Table 1. Codebook.

code	definition	example
**flow experience**		
focus of attention	no distractions, being focused, blocking out surroundings	For me personally, it's simply that I'm so completely absorbed in a work task that I can do it for quite a long time without getting very tired and completely blocking out all the other things that are going on around me […]
fun	enjoyment while performing a task	And with it, for me, also comes that it is fun.
demand-skill-balance	a task is challenging, demanding, but not overwhelming; match between challenge and own skills	[…] that it's such a perfect mix between effort in the tasks, but at the same time you can work on them well, they're not too exhausting that you can't master them at all and despair of them, and also not so boring that you completely disconnect, but rather such a relaxed work phase.
autonomy	sense of control, freedom to make decisions	That is, the flow, as I would describe it now, when snowboarding or surfing or kitesurfing, whatever, I have the bar in my hand, sort of, or board under your feet and I just ride and can decide.
clear goals	having clear objectives, defined goals in a task	What I do is that I have a clear goal in mind and can then totally focus on it and achieve it with great determination in my work in this situation.
motivation	feeling motivated, having energy for a task	Probably by flow you mean motivation and such a motivational drive that you maintain or so, that's how I would define it.

feeling of fulfillment	feeling of accomplishment and absorption when performing an activity	I am completely caught up in it.
sense of time distorted	no feeling for time passing, time seems to pass faster in flow	Somehow I forget about time, or at least I don't look at the clock, because I think, I don't need that right now.
feeling of progress	feeling of being productive, making progress	It's work that goes easily right away. So it's just flow. It's flowing. It's moving forward, constructively.
physical changes	physical processes during flow, e.g. (not) feeling hungry, cold, etc.	[…] I can sit at my desk and for me it's really like, I forget to eat, I forget to go to the bathroom, my heating system doesn't work physically anymore.
flow not known	participant never heard of “flow”, can’t define it	[…] so honestly, this term doesn't really mean anything to me now, but I can imagine what you mean by it.
**flow situations**		
strategic tasks	strategy of the start-up, planning, business plan development	Then I've definitely had that a couple of times now as well, in general when it comes to strategic planning.
creative tasks	design, social media post creation, prototype design, free writing	If I can also be a little creative in the process, or if I make a video and I can cut it and so on […]
product development	working on the product itself	Mostly when immersing in the product.
systematic tasks	searching for information, systematic processes, research work; accounting, finances	Personally, I really enjoy researching information and I'm totally absorbed in it when I'm able to gather information.
technical tasks	tasks with technological aspects	Yes, maybe a technological factor.

practical tasks	get to work, do things by oneself, produce something	So just sitting in a quiet room and designing or drawing or something like that and then maybe going to a machine and building the first prototype and stuff like that. That is a very nice experience.
no specific flow task	flow in different tasks, type of tasks doesn't influence flow directly	In the end, I don't think it's the task that matters, it's the feeling of creating something, and achieving it constantly in a positive sense.
interest	tasks that fit one's interests and abilities; that are meaningful and fun to do	Well, I would say all the things which I have much fun with. That makes it easier to stay in the flow.
learning experience	discovering new things, learning, new challenges, unknown tasks	So these are things like suddenly discovering something, seeing something, and also just being able to understand. I think for me flow is very often associated with "I understand something that I didn't understand before".
interaction	teamwork, interacting with colleagues, clients, other people	Most of the time, it's actually in a team. Not alone.
early stage of the start-up	idea of the start-up still new, in development	Um, especially in the beginning. So the first, I can't put an exact number on it, but I would say about six months it was actually a continuous flow experience, because then everything really took off and because it was really exciting and I was completely immersed in it […]
few flow in start-up context	less flow situations in start-up compared to other domains	In the workplace [before], I think I've actually experienced that almost more than I have in the start-up business.
**factors conducive to flow**		
well-being	being in a good mood, well-rested	And I have that very often when I feel well mentally […]
task variety	diversified tasks, different high requirements alternate	And from the task itself, I see in any case a certain variety. In other words, phases where it becomes more difficult. Phases where you can just rest a little

		bit mentally and put something together or so, and have a certain variety in any case.
demand-skill balance	balance between requirements and skills; not being bored, not overwhelmed	So I really think this point, as you said, with the excessive and insufficient demands, that's kind of incredibly important, that it's just neither of them.
positive feedback	affirmations, recognition from others, positive feedback from the task itself, first achievements	If you always have such small feelings of success coming from the outside, that you were mentioned somewhere, for example, or that someone approached you and even asked you about your idea.
autonomy	sense of control, freedom to make decisions	freedom to make decisions, no time limit, no constraints
control	knowing how things work, having control over the task	But from 100% control, I gain more control with every step I take. I know which direction I'm going. The.... The nebulosity is clearing and I'm getting an idea of where I'm coming out. I think that's an important aspect.
meaningfulness	belief in own idea and potential of the start-up; task is perceived as significant, meaningful	[…] when I realized, oh, that's something, where I really pursue my mission in life. So, with this I fulfill a little bit of what I want to change in the world.
clear goals	clear objectives within of a task, structure, knowing what to do	Maybe having a clear goal of what you want. Otherwise it's hard to get into it.
moderate stress	a moderate pressure or mild stress, being excited, stage fright	And the other thing is kind of a light pressure, I would say. So, not the deadline due in two hours and you're screwed if you don't make it by then, but so that it doesn't hurt yet, it' s still okay just knowing you have to do it.
learning experience	discovering new things, learning, new challenges, unknown tasks	So first of all they [= the flow situations] are a possibility to learn. I just love to become smarter.
environment	influences from the environment, weather, workplace design, external factors	And environment, for me a room where I feel comfortable […] where I can create the atmosphere.

equipment	required resources, tools, programs available	Maybe that's all it takes, easy access to tools. So that whatever it is, whether it's a machine where you can make something out or whether it's a well-filled bank account where you simply order something to try it out.
tranquility / no distractions	quiet environment	Definitely being in a quiet area.
work alone	individual work, no interactions	So I work, for example, I already did before, even before the pandemic,… I have moments where I have to be all to myself. That's definitely an important factor, or simply my privacy. I also do not want to see anyone around me.
interaction	teamwork, interacting with colleagues, clients, other people	Teamwork in general, good teamwork, can contribute a lot to this.
interest	tasks matching own interests and preferences	Well, I think it's all a kind of related to my interests and strengths, if you take it that way.
**factors inhibiting flow**		
physical limitations	physical constraints, limitations, lack of physical resources	Well, I guess like I said earlier: back pain.
overload	high demands, when one does not know what to expect, unknown task, high difficulty, challenge	[…] when I'm overloaded. That is, when I'm stuck on something and don't know how to continue.
failure	things don't work out as planned, unexpected incidents; negative feedback	One has a certain schedule or structure in the day and when for me something starts to not work properly.
lack of interest	tasks for which enthusiasm or interest is lacking, when a task is not enjoyable	Or if it's tasks that I'm not at all comfortable with, that I don't enjoy at all, and then I kind of end up stuck in front of it.

multitasking	other tasks, e.g. regular job, academic studies, private life, multiple tasks at a time	Yes, I think the typical thing is that you have something else to do that is more important or at least has a high priority, so that you can't fully focus on it. So, I was still working in the hospital in parallel for quite a while when founding the start-up, and it's obvious that when you're working there, you're not able to focus on your goal, “flowing” in your start-up.
distraction	distraction in terms of noise, other people, environmental factors	So when I notice that something triggers me from the outside, then I either have to turn off the trigger or I surrender to the trigger, but then I'm no longer in the flow and do other things in parallel.
dependence	waiting for feedback, dependency on input from others; dependency on the functioning of technical equipment	So if I have to rely on others to do the work and it's not there or it's not right, then that puts me off.
online communication / virtual meetings	lack of personal contact, lack of goal-oriented direct communication	And as I said, I don't really like these video conferences in the team, especially when you have to discuss important topics.
conflicts	disagreements with co-founders, conflicts in the private sphere that affect	[…] disagreements in the team – you also have that sometimes – this breaks the flow.
stress / pressure	stressful work phases, lack of time	So time pressure is also not at all... so it's very counterproductive for that.
**consequences of flow**		
fun	enjoyment with the work, happiness, euphoria	Like hey that was actually really super fun to do and super interesting.
motivation	be encouraged, more motivated to continue working, less doubts, more willing to take risks	Well, that's great, of course, when you're more motivated then.
energy	energetic feeling, being very awake, urge to move, physical effects	Um, yeah, I'm just totally full of energy.

satisfaction	being satisfied with what one has achieved, seeing what one has accomplished; fulfillment	In the end, I think it just felt good to get satisfying results out of it. You're just very, very satisfied with what you've come up with and you feel good about implementing it.
resilience	be less likely to be thrown off track by failures, overcoming setbacks positively	And if I then have someone who calls me and is not so polite or so, then I am much, much more resilient against it. Then I think to myself, much more "well", whereas maybe in other situations, when I had no flow at all on that day, it hits me more.
self-efficacy	be more confident, trust in your own abilities	I somehow just get more accomplished, I'm more convinced of myself that I can do things […]
better progress	work more productively, work more efficiently, faster	Yes, it was just extremely productive, extremely good. So effective, efficient. Just in terms of the use of time, that I simply managed to get a lot done in a short period of time, I was incredibly productive.
better results	results get better (not only achieved faster, but also in terms of quality)	A much clearer mind, better ideas […]
work more	no time limit, ongoing work for hours, no need for breaks	Overtime so/ yes. What does overtime mean? Forgetting the time. Well, you have to. To be honest, you don't think about it at all.
master unpleasant tasks	less procrastination, more motivation to work on unattractive tasks	[…] I still think that you avoid some things and when you're in the flow, that doesn't happen.
learning experience	continue to learn, develop further	I have learned a lot.
new ideas	creativity, working on new ideas, innovative behavior	I get good ideas then. So I suddenly find solutions for things that, if I had sat down in a different way and tried to solve them... I probably wouldn't have come up with so much.

teamwork	engage with others in one's own flow, interaction, collaboration	And because there are two of us and we don't always have a flow at the same time, we of course have an effect on each other.
perfectionism	get lost in a task, try to make it perfect	Rarely, I would have said, perhaps it leads to the fact that one is too perfectionist in something like that, but that may also be personally driven, so that one says "okay, I want to do it this way or that way now" and then somehow one has gotten stuck into it.
neglect of other tasks	forget about / don't care about needs, demands, problems other tasks	[…] because you do a lot in a very short time, because you only think about the one thing and maybe neglect other things. But still, the experience is very positive.
lack of communication	rushing ahead, taking decisions to fast, don't communicate with team members during flow	Yes, also because in my opinion flow also leads to the fact that one rushes ahead a little bit and makes premature decisions, because you are in flow and then you just call a few people or settle things that are perhaps not planned or discussed in detail yet.
focus	strong focus of attention, suppress external factors	When you're in the flow, it's just relaxed and you're so extremely focused. And I think that sometimes it's also good to be torn out of this pure focus, because then it can of course also happen that you miss some things.
**team flow experience**		
flow can be experienced in a team	participants confirm having experienced shared flow in the team, no further explanations	There are those moments when we experience flow as a team.
interaction	direct communication, active interaction, conversations	Yes, definitely just by interacting. So, the individual flow is really that you kind of sink into your figures and can block out everything around you. Of course,

		it's a different situation in the team because you have an incredible amount of interaction and it's a different way of working, a different feeling.
engage with each other	be aware of the others and their needs, strengths, habits, etc.	But it's very different because you're always interacting and therefore you're not that deep in the task because you're also focused on the other person and not just on the task.
clear shared goals	move in the same directions, work on the same objectives, have the same vision for the start-up	The fun thing is that everyone has the same vision for a topic at that moment, and that means we're all going in the same direction.
focus of attention	team members work in a focused way	One is simply more focused. You notice that very strongly when this flow experience occurs together.
individual flow	combination of individual flow experiences, can but does not have to be a joint task	So I had the feeling that in the team it's mainly about one idea sparking the other.
**team flow situations**		
creative tasks	design, social media post creation, prototype design, free writing	With design issues, it was the case.
strategic tasks	strategy of the start-up, planning, business plan development	It's a bit similar with sales. Sales itself is not an area where I get into the flow, but the development of possible sales territories and target groups is something I also find very exciting in any case.
product development	working on the product itself	So it's also super exciting when you can really imagine what the product looks like and then finally have the product in your hand at some point. That's very cool and fun, because it's really about what you want to do in the end.

interest	tasks that fit one's interests and abilities; that are meaningful and fun to do	I think that's more of a question of topics. There are things that are more fun and things that are less fun.
collaborative tasks	work together on one task	Meanwhile also when we built our website, where the four of us sat together and met up.
interaction	discussions, talking to each other, meetings	And that's how it was, that we always communicated with each other throughout the day. Person A does this, person B does that, person C and D do that together. That was a very fulfilling experience or a good feeling […]
new tasks	novel tasks, engaging in new things together	So especially when it's new things, when there's new things coming up, we're getting involved in something new at this time.
**factors conducive to team flow**		
well-being	team members in a good mood, well-rested, psychically and physically in good shape, no conflicts in the team	Just, have everyone slept well, did everyone eat well?
clear goals	clear objectives within of a task, structure, knowing what to do	So I also believe a clear goal, okay, we're now going to discuss this and that for an hour, that the task is clearly defined.
moderate stress	a moderate pressure or mild stress, being excited, stage fright	Then this, I'll call it positive stress before the launch, as I said, is definitely something that has brought us forward.
autonomy	sense of control, freedom to make decisions	And when there is a phase where we are both very excited about our plans, about our startup, get very involved in it, mentally as well, and also both have the capacity to do so, because we are both also people who still have a lot to do outside of the startup […]

positive feedback	affirmations, recognition from others, positive feedback from the task itself, first achievements	When we then took this step and also contacted everyone and also received the first feedback and then also the positive feedback, that was such a point.
meaningfulness	belief in own idea and potential of the start-up; task is perceived as significant, meaningful	And since then, however, it is there and there is also the enthusiasm, belief, inner conviction, intrinsic conviction, passion, etc. […]
personal contact	meetings in person (instead of online communication)	So I think in this case one factor was that we saw each other in person after a long time, that it was something special, so that motivated us in this case and that was simply an influencing factor.
environment	influences from the environment, weather, workplace design, external factors	And then it was also the environment. I'm very sure that it was extremely because of it. […] [In the co-working space] they have different rooms for co-working or for seminars or something like that, and the rooms are especially designed to stimulate creativity, and that really works.
tranquility / no distractions	quiet environment	Definitely being isolated from outside influences, whether it's in a co-working space, going into a team room, that you're isolated, being isolated from also electronic influencing factors, cell phone and so forth.
interest	tasks matching own interests and preferences	[…] it just depends extremely on how enjoyable the tasks are or not.
common basis	matching of prior knowledge, expertise, skills, abilities of team members, joint work on specific task is possible	And then also, what it comes down to in the end, is what competencies do the team members bring with them?
commitment	shared goals, identification, pleasure of working together	[…] if you just have a healthy environment with people who are as different as possible, but still all have a desire to work on the same topic.
team spirit	knowing each other, knowing and thus accepting each other's strengths and interests,	So there really has to be a deep fundamental trust […]

	nonjudgmental interactions, open atmosphere, supporting each other, trusting each other	
**factors inhibiting team flow**		
physical limitations	physical constraints, limitations, lack of physical resources	But I definitely think if you're stressed, then you shouldn't make important decisions, [or] when you're kind of hungry, tired.
overload	high demands, when one does not know what to expect, unknown task, high difficulty, challenge	Yes, I could imagine, I don't know exactly how you could describe it, but if you don't really get into the activity […]. Then I think it's hard to get involved. So there could also be activities where many people are not so inspired and just can't think of anything. And then I think it becomes difficult.
routine	lack of new challenges, getting used to tasks that are getting boring with time	Sometimes, when you're in the daily routine or you're not making good progress on something, on an existing issue, then I notice that it leads to fewer experiences like this.
failure	things don't work out as planned, unexpected incidents; negative feedback	Um. Yes, of course, when you realize that maybe in [city A], for example, it's going really well and in [city B] it's not going so well. That takes away the flow, or it has taken away the flow a bit, because [city B] hasn't developed as well as we expected.
lack of feedback / result visibility	progress in small steps whose result is not immediately apparent in the big picture, little feedback from the task	And I notice that it doesn't quite work that way, because we don't achieve these steps, this result in a way that is visible step by step, but rather it is somehow very fragmented.
multitasking	other tasks, e.g. regular job, academic studies, private life, multiple tasks at a time	We also have the problem, of course, that not everyone always has time. Two of our co-founders also have full-time jobs. So it's difficult, let's say, to take the whole day. But when we do, it always has a positive effect for us.

dependence	waiting for feedback, dependence on input from others; dependence on the functioning of technical equipment	I think with the website in particular it was the case that we simply couldn't continue in the meantime and first had to solve certain problems in order to implement the things further and then it could only be done by one person.
distraction	distraction in terms of noise, other people, environmental factors	Yes, noise in any case. Or anything that is simply annoying in some way. Such common disruptive factors.
online communication / virtual meetings	lack of personal contact, lack of goal-oriented direct communication	And best of all, I have to say, in personal contact with people, virtually I have never experienced that a good flow has developed, rather it was always very exhausting and stressful.
conflicts	disagreements with co-founders, conflicts in the private sphere that affect	Well, disagreements, as well. Half a year ago, there was a really critical situation, because we had disagreements in the team. That definitely killed the flow for a few weeks.
stress / pressure	stressful work phases, lack of time	So somehow, on an evening before a working day or so I think, it wouldn't have gone like that. So that you also really – yes, of course, you can also look at it negatively that it was on a weekend day – but I think we also need that that it was the whole day. Enough time, so again this no time pressure.
lack of common basis	different levels of knowledge, different assumptions; different levels of commitment to the start-up idea	When one is more convinced than the other. Let’s say when the common mission is disrupted.
**consequences of team flow**		
satisfaction	being satisfied with what one has achieved, seeing what one has accomplished; fulfillment	I think the most important thing is joy and hope.

motivation	be encouraged, more motivated to continue working, less doubts, more willing to take risks, motivate each other	And I think all the participation of the others also motivated the others again, because "oh great, now there's a totally fantastic idea from this one and that one, awesome”.
better progress	work more productively, work more efficiently, faster	Being focused and in the flow together, then you can achieve quite a lot.
better results	results get better (not only achieved faster, but also in terms of quality)	In the end, I would say, definitely a better work result […]
team spirit	growing closer as a team, trust; communication, mutual interaction	Well, at that moment, it feels like we're getting closer again in the team, I'd say, or more closely connected, that's what I would call it now, from a team perspective. And that you just feel more like a unit, or this team spirit, that we shape and are the company.
collective efficacy	have confidence in oneself as a team and the team’s skills	And I think it has this effect above all, that you get the feeling of all the things you can accomplish.
vulnerable to setbacks	being torn out of team flow even harder experience to handle for a team, difficulties in getting back to a positive state	However, if you're always in this flow mode, things are going very well, you're making progress, then you can also become dissatisfied quite quickly if small things now set you back again, for example.
focus	strong focus of attention, suppress external factors	Um, maybe if it actually goes into too much detail on specific things.
